# Evaluation of an influenza-like illness case definition in the diagnosis of influenza among patients with acute febrile illness in cambodia

**DOI:** 10.1186/1471-2334-10-320

**Published:** 2010-11-07

**Authors:** Matthew R Kasper, Thomas F Wierzba, Ly Sovann, Patrick J Blair, Shannon D Putnam

**Affiliations:** 1 U.S. Naval Medical Research Unit 2, Jakarta, Indonesia; 2 U.S. Naval Medical Research Unit No. 2, Phnom Penh, Cambodia; 3 Communicable Diseases Control Department, Kingdom of Cambodia Ministry of Health, Phnom Penh, Kingdom of Cambodia

## Abstract

**Background:**

Influenza-like illness (ILI) is often defined as fever (>38.0°C) with cough or sore throat. In this study, we tested the sensitivity, specificity, and positive and negative predictive values of this case definition in a Cambodia patient population.

**Methods:**

Passive clinic-based surveillance was established at nine healthcare centers to identify the causes of acute undifferentiated fever in patients aged two years and older seeking treatment. Fever was defined as tympanic membrane temperature >38°C lasting more than 24 hours and less than 10 days. Influenza virus infections were identified by polymerase chain reaction.

**Results:**

From July 2008 to December 2008, 2,639 patients were enrolled. From 884 (33%) patients positive for influenza, 652 presented with ILI and 232 acute fever patients presented without ILI. Analysis by age group identified no significant differences between influenza positive patients from the two groups. Positive predictive values (PPVs) varied during the course of the influenza season and among age groups.

**Conclusion:**

The ILI case definition can be used to identify a significant percentage of patients with influenza infection during the influenza season in Cambodia, assisting healthcare providers in its diagnosis and treatment. However, testing samples based on the criteria of fever alone increased our case detection by 34%.

## Background

Globally, influenza is considered one of the most important infectious diseases. It is reported that between 3 and 5 million cases of severe influenza disease occur each year [1], with estimated annual influenza-associated mortality between 500,000 and 1,000,000 cases (median case-fatality of 190 deaths per 100,000 person infected with influenza) [2,3]. Complicating the global influenza burden is the recent recognition of a novel quad-reassortment swine-origin influenza A virus which is the agent associated with the WHO declared influenza pandemic [4].

Influenza viruses are transmitted through the respiratory route [5-8] and infections vary from asymptomatic to severe, life threatening. Common clinical symptoms of influenza include fever, cough, sore throat, headache, muscle aches, nasal congestion and weakness [9]. These symptoms can be non-specific and do not easily distinguish influenza from other respiratory viral syndromes or other infectious etiologies in patients presenting for healthcare services with acute febrile illness [10]. As with any standardized syndromic disease case definition, definitions of "influenza-like illness" (ILI) vary [11-13] but typically include fever (≥ 38°C) with one or more respiratory symptoms (e.g., cough or sore throat). These clinical algorithms have been studied in children and adults as part of hospital-based or age-specific antiviral trials. These studies suggest that an ILI definition including cough has a positive predictive value (PPV) of 60% to 87% [11,14,15]. Other work has focused on influenza in hospitalized patients [16,17] and in national surveillance activities [18]. The majority of these efforts have focused on populations from developed countries, whereas information on the predictive values of ILI symptoms in developing countries is limited.

Building upon previous influenza surveillance results from rural Cambodian patients [19], we evaluated a commonly used ILI definition, document fever and cough or sore throat as a predictor of influenza disease. As previous studies have suggested limiting the use of clinical predictors for influenza to the influenza season [15], the sampling included all eligible patients enrolled during a single Cambodian influenza season.

## Methods

### Study site and population

In December 2006, a clinic and hospital-based acute febrile illness surveillance was implemented at nine Cambodian government medical clinics. Five of these sites were located in Operational District A (peri-urban) and four were in Operational District B (rural). All participating field sites were within 50 kilometers of Phnom Penh in south-central Cambodia. Patients were recruited by study site staff if they had a recorded temperature ≥ 38.0°C lasting at least 24 hours but not greater than 10 days, were two years of age or older, and, after medical examination, had no obvious source of infection. A healthcare provider in each clinic obtained written informed consent, administered a pre-tested enrollment questionnaire, performed a medical examination and collected clinical specimens per study protocol[19]. Influenza-like illness was defined according the WHO guidelines, which included, documented fever (≥ 38.0°C) and cough or sore throat. For this study, only patients enrolled I the surveillance for acute febrile illness from July 2008 through December 2008 were included for analysis; corresponding to influenza season in Cambodia.

### Specimen Collection

For each enrolled patient, one throat and one nasal swab were collected. For nasal swabs, a dry polyester swab was inserted into the nostril parallel to the palate, slowly withdrawn, and placed in a vial containing 2 - 3 milliliters of virus transport medium (VTM). For throat swabs, both tonsils and the posterior pharynx were swabbed vigorously, and the swab placed in 2 - 3 milliliters of VTM. All inoculated vials were kept at 4°C until transported between 24 and 72 hours after collection to the Naval Medical Research Unit No. 2 (NAMRU-2) located at the campus of the Cambodian National Institute of Public Health (NIPH).

### Laboratory testing

Ribonucleic acid (RNA) was extracted from nasal and throat swabs using QIAamp viral RNA mini kits (QIAGEN, Hilden, Germany) following the manufacturer's instructions and stored at -70°C. The influenza virus genome was detected using a reverse real-time PCR (rRT-PCR) assay developed to detect influenza A/H1, A/H3, A/H5, and B virus subtypes. Real-time assays were developed at the Centers for Disease Control and Prevention (Atlanta, GA, USA). Real time assays and sequence information are available upon request from Dr. Steve Lindstrom under the terms of a material transfer agreement. One-step rRT-PCR was performed in a final volume of 25 μl containing 5 μl of extracted RNA, 12.5 μl of buffer mix and 0.5 μl Superscript III/Platinum Taq-Enzyme mix, 20 units of RNAse-out (Invitrogen, Carlsbad, CA, USA), 0.8 μM for each primer and 0.2 μM of probe. The Rotor-Gene 6000 real-time thermocyler (Corbett Life Science, Sydney, Australia) was used for all PCR reactions. The thermocycling parameters for all targets consisted of 50°C for 30 minutes, 95°C for 2 minutes, and 45 cycles with 95°C for 15 seconds, 55°C for 30 seconds (US CDC, Atlanta, GA, USA).

### Statistical Analysis

All data was double-data entered into MS Access (Microsoft Inc., Redmond, WA, USA). Data was imported into SAS v9.1 (SAS, Cary, NC), which was used for all statistical analyses. The definition of ILI (fever and cough or sore throat) was analyzed to determine sensitivity, specificity, positive predictive valve (PPV) and negative predictive value (NPV) comparing patients with and without laboratory evidence of influenza virus. Sensitivity was defined as the probability of having the ILI given laboratory-confirmed influenza; specificity was defined as the probability of not having the ILI when the patient does not have laboratory-confirmed influenza. The PPV was the probability of having laboratory-confirmed influenza when ILI was present; NPV is the probability of not having laboratory-confirmed influenza when ILI was not present[15]. Categorical data was analyzed by the use of either Chi-Square (expected cell frequency > 5) or Fisher's Exact Test (expected cell frequency ≤5). Continuous data were assessed for normality and if normally distributed, parametric statistics were used. If the data was non-normally distributed, then non-parametric testing was used. All statistical tests were two-tailed and significance was defined as p < 0.05.

### Ethical Considerations

Eligible subjects were voluntarily enrolled in accordance with an Institutional Review Board protocol approved by U.S. NAMRU-2 and the National Ethics Committee of the Royal Kingdom of Cambodia, Ministry of Health.

## Results

From July 2008 through December 2008, a total of 2,639 febrile patients were enrolled. The median age of enrolled patients was 11 years (Interquartile Range (IQR) 6 - 25) and males accounted for 52.9% of enrolled patients. The median day of patient presentation was at 3 days of fever duration (IQR 3 - 4) with a median temperature of 39.0 (IQR 38.5 - 39.5). A more detailed description of this population has been described elsewhere [19].

Among the enrolled patients, the prevalence of laboratory-confirmed influenza was 33.5% (884) and the proportion of patients with ILI was 62.2% (1,652). Based on these values, the sensitivity and specificity of the ILI cases definition to predict disease was 73.8% (652/884) and 43.0% (755/1755), respectively. The PPV was 39.5% (652/1652) and NPV was 76.5% (755/987). Among enrolled patients, laboratory testing of patients using the criteria of only fever increased the number of detected cases from 652 to 884, a 34% increase.

Of the 884 laboratory-confirmed influenza cases, 69% (608) and 31% (276) had influenza A and B types, respectively. (Table 1) Among patients with influenza A and B, ILI sensitivity was 76.0% (462/608) and 68% (190/276), respectively suggesting that the ILI case definition was 1.4 times (95% confidence interval (CI): 1.03 to 1.99, p = 0.025) more sensitivity in detecting influenza A as compared to than influenza B cases. Sensitivity did not differ between influenza H3 and H1 types.

**Table 1:** Patients (N = 2,639) presenting with influenza-like illness and laboratory confirmed Influenza type and subtype by clinical presentation of Influenza-like Illness (ILI).

Influenza Status	ILI	NO ILI	Total
Influenza (overall)	652 (73.8%)*	232	884
A †	462 (76.0%)	146	608
A/H1‡	78 (74.2%)	27	105
A/H3	383 (76.3%)	119	502
A/H5	1 (100%)	0	1
			
B	190 (68.8%)	86	276
Negative	1000	755	1,755

* n (% positive for ILI)

† Odds ratio = 1.4 (95% confidence internals: 1.03 to 1.99, p = 0.025) for frequency of ILI by influenza A (76.0%) and influenza B (68.8%) types

‡ ILI for H1 (74.2%) vs. H3 (76.3%), p = .66

When stratified by age groups (young children (ages 2-6), school aged children (ages 7-18) and adults (>18 years of age)), there was no detected statistically significant difference for ILI sensitivity. However, there were noted statistically significant differences by age group for specificity (p < .0001), PPV (p = .01), and NPV (p < .0001). (Table 2) For all three values, analysis suggested that adults had higher specificity (53%), NPV (90%), and lower PPV (21%) as compared to subjects in the younger age groups.

**Table 2:** Sensitivity, Specificity, positive and negative predictive value for a case definition of Influenza-like Illness by presence or absence of laboratory-confirmed influenza virus infection.

Age (years)	2 to 6	7 to 18	>18	p-value
Sensitivity	76 (335)*	73 (401)	68 (148)	.18
Specificity	32 (470)	37 (487)	53 (798)	<.0001
Positive Predictive Value	44 (576)	49 (599)	21 (476)	.01
Negative Predictive Value	66 (229)	63 (289)	90 (471)	<.0001

* Percent (number tested)

When stratified by month, the PPV was lowest in July for all age groups, peaking in October, and declined thereafter (Table 3). While the monthly pattern was the same for all, the PPV statistically differed by age group for September through December. PPVs during these months were lowest for adults, although 2 to 5 years olds had lower rates than 7 to 18 year olds in December. Over the entire period of study the PPVs for age groups 2 to 6, 7 to 18, and >18 years was 44%, 49% and 21% (p < .0001), respectively. Again, adults had the lowest overall PPV.

**Table 3:** Positive predictive value of having influenza virus given ILI symptoms stratified by age group and enrollment month.

Age (years)	2-6	7-18	>18	p-value
July	4	16	9	.10
August	22	24	19	.78
September	60	59	26	<.0001
October	65	73	33	<.0001
November	53	52	20	<.001
December	9	32	7	<.0001
OVERALL	44	49	21	<.0001

**Figure 1:**
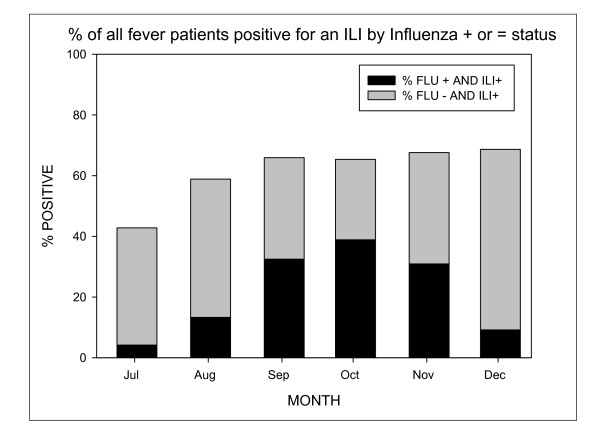
**Among patients with an acute febrile illness, the percentage of patients with ILI that were positive or negative for influenza infection**.

From July to December 2008, ILI was documented in 43% to 68% of enrolled acute fever patients (Figure 1) per month while the prevalence of influenza ranged from 9.9% in July to a peak of 59.4% in October. ILI criteria was met most often in December (68%), when the prevalence of influenza was only 9.7%.

## Discussion

Previous studies have reviewed the definition of ILI and symptoms most commonly associated with influenza disease, with most studies using hospitalized patients [9,11-13]. This analysis evaluated the ability of an ILI-defined syndrome to correctly identify patients with a laboratory-confirmed influenza infection in Cambodia patients seeking treatment for acute febrile illness. We found that the ILI case definition was more sensitive for patients infected with influenza A than influenza B, possibly reflecting milder disease among those infected influenza B as described in previous studies [20,21]. The PPV of the ILI definition changed by month as changes in the prevalence of influenza infections occurred. Hence the PPV appeared to peak in October, at the peak of the influenza season. The sensitivity of ILI case definition did not appear to vary with age. However, the specificity appeared highest in adults relative to children suggesting that adults were less likely to present with cough or sore throat than children possibly due to acquired immunity to respiratory agents that might induce ILI symptoms.

Laboratory testing patients only with ILI as enrollment criteria would have identified 74% of the influenza cases among enrolled patients. The definition of ILI for surveillance purposes is sufficient as a testing strategy to monitor seasonal influenza circulation and emerging influenza strains. The ILI definition did not detect 26% of cases in our clinic-based surveillance and may be lacking for clinical practice and treatment guidelines without laboratory testing to support the diagnosis.

The study enrollment criteria of reported fever duration for at least 24 hours does differ from the WHO ILI definition that has no defined duration of fever. It is possible that few patients seeking health care very early in their illness may not meet study enrollment criteria and therefore not been enrolled. However, the median day of fever reported among patients upon enrollment was three days with an IQR of 3-4 days, suggesting that most Cambodian patients were unlikely to seek care until well into their disease. In addition, further work is needed to understand the healthcare seeking behavior of this study population.

From August 2008-December 2008, the percentage of febrile patients with ILI stayed constant study despite a varying prevalence of influenza, suggesting that other pathogens are contributing to febrile respiratory illness in Cambodia. In addition to influenza virus, recent studies have identified other respiratory pathogens such as human metapneumovirus [22], respiratory syncytial virus, and parainfluenza virus 3 [23], adenovirus and rhinovirus from patients in developing countries; Further studies are required to determine the contribution of non-influenza respiratory pathogens among Cambodians presenting with ILI. The pathogens endemic (e.g. dengue virus and malaria) to this region and the different age groups they are most commonly detected in must also be considered.

Previous studies have determined that the best use of predictors is during the influenza season [15]. However, these studies have focused on seasonal influenza in developed nations and compared to these studies, the PPV of ILI identified in Cambodia was lower, even at the peak period of seasonal transmission. The range of the monthly prevalence over the course of the influenza season had considerable impact on the predictive values and suggest that even within an influenza season the use of clinical predictors may have limitations.

## Conclusions

This study allowed us the unique opportunity to evaluate an ILI definition among all patients with an acute fever regardless of associated symptoms. Based on our results, the ILI case definition can be used to identify a significant percentage of patients with influenza infection during the influenza season in Cambodia. However, testing samples based on the criteria of fever alone increased our case detection by 34%. Surveillance for acute fever provided a significant increase in the number of influenza patients identified and should be considered when determining the prevalence of influenza in a population or when using clinical case definitions to study the transmission of influenza among populations or study groups. While the ILI definition may indeed identify the majority of cases in a population presenting for treatment, it may underestimate the true burden of influenza disease among persons in resource-poor regions.

Those patients without respiratory symptoms who nonetheless tested positive for influenza are a reflection of the difficulty of its clinical diagnosis, a difficulty compounded by pathogens endemic to the region presenting as acute undifferentiated febrile illness such as dengue, malaria, typhoid fever, and other respiratory pathogens.

## Competing interests

The authors declare that they have no competing interests.

## Authors' contributions

MRK - Data analysis, writing manuscript. TFW - Data analysis, writing manuscript. LS - Review of manuscript. PJB - Study design, review and corrections of manuscript. SDP - study design, data analysis, writing manuscript. All authors read and approved the final manuscript.

## Pre-publication history

The pre-publication history for this paper can be accessed here:

http://www.biomedcentral.com/1471-2334/10/320/prepub
